# Orthogeriatric co-management for proximal femoral fractures. Can two additions make a big difference?

**DOI:** 10.1186/s12891-020-03392-1

**Published:** 2020-06-11

**Authors:** Maic Werner, Olaf Krause, Christian Macke, Lambert Herold, Alexander Ranker, Christian Krettek, Emmanouil Liodakis

**Affiliations:** 1 Trauma Department, Hannover Medical School (MHH), Carl-Neuberg-Str. 1, 30625 Hannover, Germany; 2 Institute for General Medicine, Hannover Medical School (MHH), Carl-Neuberg-Str. 1, 30625 Hannover, Germany

**Keywords:** Proximal femoral fracture, Orthogeriatric co-management, Co-management, Geriatric, Satisfaction, Length of stay, Time to surgery, EQ-5D

## Abstract

**Background:**

Proximal femoral fractures are a major socioeconomic burden and they occur mainly in geriatric patients. High mortality and complication rates are reported. To reduce the mortality and morbidity of these patients, co-management with geriatricians has been recommended. Most previous studies have focused on relatively comprehensive care models. Models with only a few additions to the usual care have not been extensively evaluated.

**Methods:**

This retrospective observational study included all patients aged ≥70 years (mean age: 84.5 ± 7.1 years, 70% women) with an isolated proximal femoral fracture treated surgically in our institution from May 2018 to October 2019. In the first 9 months, patients were treated with the usual care (control group, *n* = 103). In the second 9 months, patients were treated with our multidisciplinary care model (intervention group, *n* = 104), which included the usual care, plus: (1) one multidisciplinary ward round per week and (2) one “elective” operation slot per day reserved for proximal femoral fractures. Baseline characteristics and outcome measures of the hospital stay were extracted from electronic health records. A 3-month follow-up was conducted by phone.

**Results:**

Baseline characteristics were comparable between groups (*p* > 0.05). The hospital stay was shorter in the intervention group than in the control group (7.8 ± 4.3 vs. 9.1 ± 4.5; *p* = 0.022). The intervention reduced the waiting time for surgery by more than 10 h (intervention: 25.4 ± 24.5 vs. control: 35.8 ± 34.1 h; *p* = 0.013). A structured phone interview was not performed in 30.9% of the cases. The model reduced the overall dissatisfaction rate by more than half (12.9% vs. 32.4%; *p* = 0.008). On the other hand, the groups had similar perioperative complication rates (25% vs. 24.3%; *p* > 0.9999) and mortality (4.8% vs. 3.9%; p > 0.9999) and they remained similar at the 3-month follow-up (complications: 20.3% vs. 17.6% *p* = 0.831, mortality: 18.2% vs. 15.0% *p* = 0.573).

**Conclusion:**

We found that two additions to the usual proximal femoral fracture regimen could significantly improve the overall satisfaction rate, reduce the length of hospital stay and shorten the waiting time for surgery. In contrast to previous studies, we observed no significant improvements in complication or mortality rates. Further changes in the standard care might be needed for this purpose.

## Background

The socioeconomic burden of proximal femoral fractures is rising with increases in the aging population in modern societies. In Germany, approximately 162,000 proximal femoral fractures were treated in 2017 [1]. The incidence is expected to rise in the future [2] and consequently the costs [3]. Over a lifetime, 16–18% of white women and 5–6% of white men are expected to experience a proximal femoral fracture [4].

In addition to the high incidence and costs, the outcome of these patients is poor. Inpatient complication rates are nearly 20% [5] and the 1-year mortality is 25% [6]. Many patients experience a decline in their performance of activities of daily living (ADL) and they do not regain their pre-fracture mobility level [7]. Consequently, 12 to 15% of patients aged > 65 years move to a nursing home within 6 months of their discharge from the hospital [8].

These findings indicated that there is a need for improvement in the care of proximal femoral fractures in older patients. One approach is to reduce the waiting time for surgery. Current guidelines recommend that surgeons perform surgical treatment for proximal femoral fracture within 24 h of injury, because observational studies have suggested better outcomes can be achieved within this time frame. Earlier surgery was associated with shorter hospital stays, shorter pain durations [9], fewer complications and lower mortality rates [10].

Moreover, due to many comorbidities and an increased age [11] some hospitals combine orthopedic and geriatric expertise in the treatment of these patients. In the traditional care the patients are treated by an orthopedic surgeon and geriatric consultation is only performed on request [12]. Basically, there are 3 different models of orthogeriatric co-management described in the current literature. Firstly, patients can be treated mainly by orthopedic surgeons with routine consultation of a geriatrician. While some studies [13, 14] were able to show an improvement with regard to mortality this effect was not visible in all studies [15]. Secondly, patients can also be treated mainly at a geriatric unit with orthopedic consultation. The meta-analysis of Moyet et al. [16] favors this treatment concept with regard to reducing mortality. In the third mostly performed model the patients are managed together by an orthopedic surgeon and geriatrician. Whether mortality can be reduced with such a model is not clear [16–19]. However, Rapp et al. demonstrated recently in an evaluation of health insurance data the clear benefit of orthogeriatric co-management with regard to mortality [20]. Additionally, orthogeriatric treatment can result in increased and reduced complication rates [14, 21]. Multidisciplinary treatment is often followed by additional changes in treatment protocols for geriatric patients. These treatment protocols can help to reduce costs, complications and mortality [22–24]. There is still much uncertainty which of the above mentioned treatment models is most beneficial for this patient collective and how often multidisciplinary treatment is needed [24, 25].

Most previous studies investigated the effects of relatively comprehensive care models with daily interdisciplinary management and changes in geriatric treatment protocols. Often, a major change like that is difficult to implement into clinical practice, whereas the integration of only a few changes can be more feasible. This study aimed to determine whether two additions to the usual orthopedic care could improve process and outcome measurements at the hospital stay and at a follow up of at least 3 months after surgery. The main focus was on a reduction of TTS and complication and mortality rates.

## Methods

For this retrospective observational study we reviewed the charts of all patients aged ≥70 years that underwent surgery for proximal femoral fractures at our institution (Level I trauma center) between May 2018 and October 2019. Patients with concomitant fractures or injuries that needed treatment and prolonged mobilization were excluded. Further, we excluded all in-patient falls resulting in a proximal femoral fracture. Periprosthetic fractures were excluded. If patients had a second fracture within the study period, one of the admissions was randomly excluded.

This cohort was divided into two groups. The control group received no orthogeriatric co-management (1 May 2018–31 January 2019). The intervention group received multidisciplinary orthogeriatric co-management (1 February 2019–31 October 2019).

Two major changes distinguished the intervention group from the control group:

1. In 1 out of 3 available orthopedic operation rooms, one spot in the operation list was reserved for a proximal femoral fracture surgery. The goal was to reduce the surgical delay and perform proximal femoral fracture surgeries within 24 h of injury (when possible).
2. Weekly multidisciplinary rounds were introduced. The team consisted of at least one fellowship-trained geriatrician, one fellowship-trained orthopedic trauma surgeon and an orthopedic resident.

Both changes were initiated on 1 February 2019. We aimed to improve important outcome parameters with minimal changes (no significant reduction in elective cases, no significant increase in personal resources), compared to the usual care. In the usual care, patients were admitted from the emergency department to our trauma ward. They were visited daily by a resident in trauma surgery. The patients underwent surgery at the earliest time that the operation room and the required staff were available. Consultation was available from the Departments of Internal Medicine and Geriatric Medicine when specially requested. Patients were assessed on the risk of developing a pressure sore and preventive actions were taken, if needed. After surgery, patients received physical therapy on weekdays until discharge and full weight bearing was usually allowed for all patients. Patients with platelet aggregation inhibitors were operated without any change in their medications. Medication with direct oral anticoagulants (DOAC) was stopped for 24 h before surgery and given again at the first postoperative day. Vitamin K anticoagulants were also stopped before surgery and bridged with low molecular heparins. To accelerate the time to surgery, Vitamin K was supplied and the INR was thoroughly checked. A restrictive transfusion regimen was used. All patients with a Hb ≤ 6 g/dl received transfusions. Patients with 6 g/dl < Hb ≤ 8 g/dl received transfusions if symptomatic or by known cardiovascular diseases. Patients with a Hb > 8 g/dl did not receive transfusions. Patients were discharged as soon as they were medical stable and care was ensured. Frequency of physiotherapy, guidelines for mobilization after surgery, prevention of pressure sores, surgical technique, anesthesia, discharge, blood transfusion or bridging of anticoagulation did not change between the groups.

### Outcome parameters

We extracted baseline parameters measured at admission from electronic health records. These parameters included age at the time of the surgery, sex, fracture type, surgical technique, comorbidities evaluated with the Charlson Comorbidity Index (CCI) [26] and the American Society of Anesthesiologists risk classification (ASA) score, residential setting, grade of care prior to hospitalization, walking aids, Parker Mobility Score [27] and the Almelo Hip Fracture Score (AHFS) [28]. The Parker Mobility Score was calculated using the walking aids and distance. In the German health care system, there are 5 grades of care, displaying the need for care. Furthermore, we extracted data from medical records on the operation duration, length of hospital stay (LOS), time in the intensive care unit (ICU), amount of blood transfused, time to surgery (TTS), place of discharge, mobilization during the hospital stay, mobility at discharge and complications and mortality that occurred during the hospital stay. Complications were defined as pressure sores, urinary tract infections, acute kidney injury, gastrointestinal bleeding, ileus, pneumonia, myocardial infarction, pulmonary embolism, thrombosis, stroke, implant failure, luxation of implant, wound infection and delirium.

At least 3 months after surgery, we performed follow ups. We collected data by conducting a questionnaire, either at our outpatient clinic or over the phone. When patients could not answer the questions adequately, due to cognitive impairment, we asked the relatives or legal representatives to respond to the questions. This questionnaire included questions on mobility, quality of life, based on the EQ-5D-3 L questionnaire [29], degree of pain in the region of the fracture (based on a numeric rating scale from 0 to 10), the patient’s residential setting, changes in the grade of care, complications and re-admissions to the hospital. For complications and readmissions the date was questioned. Additionally, we asked patients whether they were satisfied with the care received. Satisfaction was measured dichotomous with the categories: satisfied vs. not satisfied. A dedicated questionnaire for this item was not used. When patients died before contacting them, we recorded only the date of death.

### Statistics

If values missed for a measurement, the patients were excluded from the calculation of this measurement. Continuous variables were checked for normal distributions with the Shapiro-Wilk test and expressed as the mean ± standard deviation (SD). For normally distributed data, the Student’s t-test was used to compare groups. For non-parametric continuous data, the Mann-Whitney-U test was used to compare groups. Categorical variables were expressed as the number and percentage. The Chi^2^-Test and the Fisher’s exact test were used to evaluate differences in categorical values between groups.

A two-tailed *p*-value < 0.05 was considered statistically significant. All statistical analyses were performed with the SPSS program (SPSS 26.0, SPSS Inc., Chicago, IL, USA).

## Results

In our orthopedic clinic, the total numbers of operations per month did not differ significantly between groups (control: 264.7 ± 16.6 vs. intervention: 252.4 ± 10.3; *p* = 0.079). Thus, the reservation of one spot on the operation list had no significant negative influence on the number of elective operations. We did not require new resources for our co-management program. The geriatrician involved was in the General Medicine Department at our clinic.

**Fig. 1:**
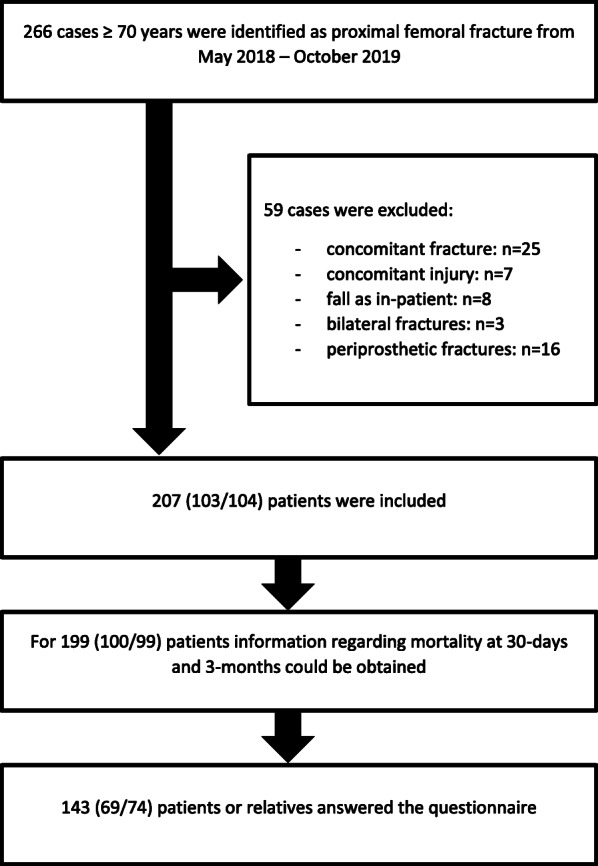
Patient inclusion

During the time of the study a total of 266 cases aged ≥70 years were identified as having surgery for a proximal femoral fracture at our institution. Two hundred seven met our inclusion criteria and 59 were excluded (Fig. 1). Of these, 103 were treated with the usual care and 104 were treated with the co-management model.

### Baseline characteristics

The cohort had a mean age of 84.5 ± 7.1 years and 70% were women (Table 1). Most patients had intertrochanteric fractures. Among all patients, two-thirds of patients had an ASA-Score of 3 or more, 48.5% of patients had already a grade of care at admission and 36.2% were living in a nursing home. Less than 50% of patients could walk without an aid, but the co-management cohort was slightly more immobile. The mean Parker mobility score was 7 ± 2.1. The groups had similar risks of early mortality, consistent with the similar AHFSs.

**Table 1:** Baseline characteristics of the control and co-management (intervention) groups

Characteristic	Control group	Intervention group	p
Patients, n	103	104	
Age, y	84.73 ± 6.66	84.19 ± 7.6	0.731
Women	72 (69.9)	73 (70.2)	> 0.9999
Fracture type			
Femoral neck	43 (41.7)	48 (46.2)	0.576
Intertrochanteric	54 (52.4)	55 (52.9)	> 0.9999
Subtrochanteric	6 (5.8)	1 (1)	0.065
Surgical technique			
Arthroplasty	38 (36.9)	40 (38.5)	0.886
Dynamic hip screw	20 (19.4)	26 (25)	0.404
Proximal femoral nail	45 (43.7)	37 (35.6)	0.257
Locking Plate	0 (0)	1 (1)	> 0.9999
CCI	1.79 ± 1.96	1.71 ± 1.49	0.743
ASA Score			0.813
1–2	32 (31.1)	35 (33.7)	
3	63 (61.2)	63 (60.6)	
≥ 4	8 (7.8)	6 (5.8)	
AHFS (risk of 30-day mortality)			0.609
Low risk (< 10)	66 (64.7)	64 (62.7)	
Medium risk (10–12)	29 (28.4)	27 (26.5)	
High risk (> 13)	7 (6.9)	11 (10.8)	
Residential setting			0.930
At home (independent)	43 (41.7)	45 (43.3)	
At home with help	23 (22.3)	21 (20.2)	
Nursing home	37 (35.9)	38 (36.5)	
Grade of care			0.563
0–1	60 (59.4)	53 (52.5)	
2	17 (16.8)	15 (14.9)	
3	13 (12.9)	19 (18.8)	
4–5	11 (10.9)	14 (13.9)	
Walking aids			
None	51 (50.5)	45 (44.1)	0.400
Stick/crutches	4 (4)	4 (3.9)	> 0.9999
Walking frame	43 (42.6)	47 (46.1)	0.672
Wheelchair	2 (2)	4 (3.9)	0.683
Bedridden	1 (1)	2 (2)	> 0.9999
Parker Mobility Score	7.24 ± 1.96	6.72 ± 2.28	0.129

Values are the number of patients (%) or the mean ± SD, unless indicated otherwise

### In-hospital outcome parameters

**Fig. 2:**
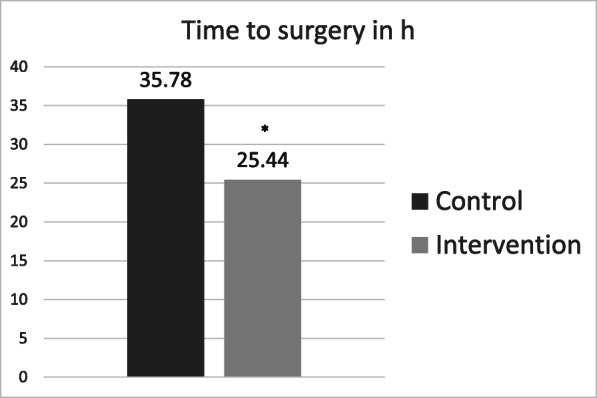
Mean time to surgery (h). * indicates a significant reduction *p* < 0.05

**Fig. 3:**
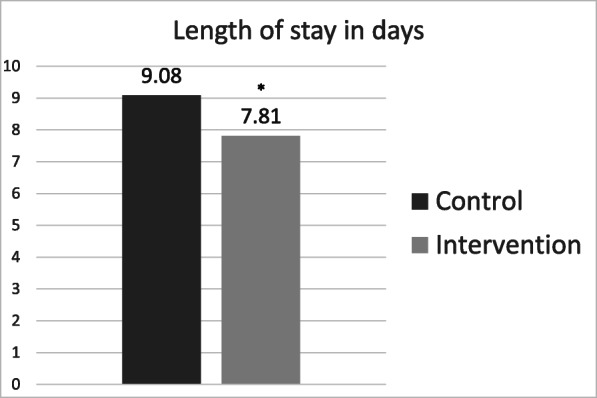
Mean length of stay (days). * indicates a significant reduction p < 0.05

The intervention group underwent surgery about 10 h earlier than the control group (35.8 ± 34.1 vs. 25.4 ± 24.5 h; *p* = 0.013, Fig. 2). Surgery was performed during regular working hours (7:30–18:00) in 25 (24.3%) patients in the control group and 33 (31.7%; *p* = 0.279) patients in the intervention group. All other patients underwent surgery during night shifts (18:00–7:30) or on the weekend. In addition, patients in the co-management cohort were discharged significantly earlier (LOS = 7.8 ± 4.3) than the control cohort (LOS = 9.1 ± 4.5 days; *p* = 0.022, Fig. 3).

The number of patients receiving blood transfusions (control: 25 (24.3%) and intervention: 21 (20.2%); *p* = 0.507) and amount of blood transfused was similar in both groups (0.63 units/patient). The groups had similar operative times and length of stay in the intensive care unit. Mobilization at hospital discharge was poor in both cohorts. Over 50% of patients could not walk at discharge (control: 46.5% vs. intervention: 57.6%; *p* = 0.155).

Delirium was the most common complication (40.1%). Most patients received general anesthesia for surgery; spinal anesthesia was administered to only 8.7% of patients in the control group and 12.5% (*p* = 0.5) in the intervention group. Except delirium, 24.6% had at least one complication (*p* > 0.9999). Of these patients 2 (1%) experienced an implant related complication (1 hemiarthroplasty dislocation and 1 DHS cut-out), both in the control group (*p* = 0.246). A total of 9 patients died during the hospital stay.

The overall dissatisfaction was more than halved after implementing our co-management program (dissatisfaction rates: control: 32.4% vs. intervention: 12.9%; *p* = 0.008) (Table 2).

**Table 2:** Outcome parameters in the intervention and control groups during the hospital stay

Outcome	Control group	Intervention group	p
TTS, h	35.78 ± 34.11	25.44 ± 24.45	**0.013**
LOS, days	9.08 ± 4.53	7.81 ± 4.29	**0.022**
Surgery time, min	72.24 ± 31.71	69.13 ± 33.52	0.368
Time in ICU, hours	19.27 ± 32.14	23.29 ± 41.29	0.725
Amount of blood transfused, units	0.63 ± 1.29	0.63 ± 1.59	0.526
Place of discharge			
Home	6 (5.8)	9 (8.7)	0.593
Early geriatric rehabilitation	70 (68.0)	65 (62.5)	0.466
Nursing home	23 (22.3)	24 (23.1)	> 0.9999
In-house transfer	0 (0)	1 (1)	> 0.9999
Death	4 (3.9)	5 (4.8)	> 0.9999
Walking aids			
None	0 (0)	1 (1)	> 0.9999
Stick/crutches	12 (12.1)	11 (11.1)	> 0.9999
Walking frame	41 (41.4)	30 (30.3)	0.138
Wheelchair	40 (40.4)	52 (52.5)	0.117
Bedridden	6 (6.1)	5 (5.1)	> 0.9999
Parker Mobility Score	3.59 ± 1.2	3.54 ± 1.23	0.352
Delirium	42 (40.8)	41 (39.4)	0.888
Anesthesia			0.5
General	94 (91.3)	91 (87.5)	
Spinal	9 (8.7)	13 (12.5)	
Complications except delirium	25 (24.3)	26 (25.0)	> 0.9999
Mortality	4 (3.9)	5 (4.8)	> 0.9999
Overall dissatisfaction	22 (32.4)	9 (12.9)	**0.008**

Values are the number of patients (%) or the mean ± SD, unless indicated otherwise.

*TTS* time to surgery; *LOS* length of hospital stay; *ICU* intensive care unit

### Short-term follow-up parameters

We contacted 143 (69.1%) patients or relatives for the follow up questionnaire, including 69 (67%) in the control group and 74 (71.2%) in the intervention group. Among these, 49% of patients responded directly to the questions and 51% required relatives to respond for them. The two main reasons for missing the phone interview were death and a change of phone number. Regarding the mortality at 3 months we were able to get information from 199 patients. The follow-up period varied from 3.02 to 19.2 months (13.39 ± 2.54 (control) vs. 6.07 ± 1.95 (intervention)).

The short-term mobility after the proximal femoral fracture was not different between groups: 21% could walk without an aid, 49% walked with an aid and 30.1% could not walk at all. Nevertheless, the quality of life measured with the EQ-5D index was slightly better in the co-management group (0.41 ± 0.3 vs. 0.46 ± 0.3; *p* = 0.38). Pain in the hip region was rated a bit higher in the co-management group (2 ± 2.7) than in the control group (1.7 ± 2.6; *p* = 0.336). Among all patients, 52.2% received an increased grade of care after the proximal femoral fracture compared to their grade of care at admission and 13.3% moved to a nursing home.

The overall 30-day mortality rate was 9.5%. Within 3 months after proximal femoral fracture surgery, 15 (15.0%) and 18 (18.2%) patients died in the control and intervention groups, respectively (*p* = 0.573). Among the remaining patients, 12 (17.6%) and 15 (20.3%) had at least one complication in the control and interventions groups, respectively (*p* = 0.831). Of these patients 3 (4.4%) had implant related complications in the control group and 5 (6.8%) in the intervention cohort (*p* = 0.721) (4 arthroplasty dislocations, 2 cut-outs of a DHS and 2 cut-outs of a proximal femoral nail). These complications led to a 10.6% re-admission rate, due to surgical or medical problems, within 3 months (Table 3).

**Table 3:** short-term outcome parameters in the control and intervention groups

Outcome	Control group	Intervention group	p
Walking aids			
None	14 (20.3)	16 (21.6)	> 0.9999
Stick/crutches	5 (7.2)	4 (5.4)	0.739
Walking frame	27 (39.1)	34 (45.9)	0.499
Wheelchair	23 (33.3)	16 (21.6)	0.135
Bedridden	0 (0)	4 (5.4)	0.121
Parker Mobility Score	5.75 ± 2.27	5.65 ± 2.52	0.993
EQ-5D index	0.41 ± 0.3	0.46 ± 0.3	0.38
Pain in hip region	1.68 ± 2.55	2.04 ± 2.66	0.336
Increased grade of care	36 (55.4)	34 (49.3)	0.494
Residential setting			0.76
At home (independent)	21 (30.4)	19 (25.7)	
At home with help	21 (30.4)	22 (29.7)	
Nursing home	27 (39.1)	33 (44.6)	
Complications within 3 months	12 (17.6)	15 (20.3)	0.831
Re-admission within 3 months	5 (7.4)	10 (13.5)	0.282
Mortality within 3 months	15 (15.0)	18 (18.2)	0.573

Values are the number of patients (%) or the mean ± SD, unless indicated otherwise

## Discussion

This study showed that two additions in proximal femoral fracture care could significantly reduce the LOS and TTS. Moreover, with these changes, a larger number of patients was satisfied with the treatment. Nevertheless, the changes did not significantly impact mortality or complication rates during the hospital stay or after a 3-month follow-up.

While detecting medical problems and preventing complications is one of the main tasks of the geriatrician in an orthogeriatric setting, Coventry et al. [30] showed higher complication rates after involvement of a geriatrician. This increase is explained by a better detection of complications. A better detection of complications may have prevented this study from showing a reduced complication rate. However, if studies showed reduced complication rates, mostly the least harmful complications were reduced [31, 32]. Delirium is common in geriatric patients after surgery for proximal femoral fracture [32, 33]. When low rates of delirium are presented it has to be questioned whether the hypoactive form of delirium is adequately represented [34, 35].

It has been reported that orthogeriatric co-management can reduce the mortality of geriatric patients with proximal femoral fracture [20]. But there is still controversial discussion which model is most effective [16]. Treating patients on an orthopedic ward with routine consultation of a geriatrician, as performed in this study, has already been found effective with regard to reducing mortality [13, 14]. However, Moyet et al. [16] could only show a slightly and not significant improved mortality for this co-management model. It has to be questioned if treating patients at a geriatric ward with orthopedic consultation would not be more effective. Besides the setting of the co-management, the frequency of consultation seems to play an important role. Only one consultation per week, as in our study, seems to be not enough for reducing mortality [36], whereas daily consultation was able to show improvements [13]. Integrating orthogeriatric care can be accompanied by a learning phase in which the outcome may be worse than before [37]. Such a transition phase may contribute to our findings.

Not only the orthogeriatric co-management model affects mortality and complication rates, but also other process aspects. A reduction of TTS and LOS is a common finding after implementation of orthogeriatric treatment [38]. With the implementation of one reserved operation slot per day we aimed to reduce the TTS and consequently the mortality and complication rates [9, 10]. While we managed to reduce the TTS, we were not able to show a reduced mortality, as expected. The association between TTS and mortality is discussed controversially. Some studies discuss cut-offs that are higher than our TTS in the control group for a better outcome [39, 40]. Reducing the TTS further, as in our study, would therefore be without effect. Other studies indicate that the shown reduction in mortality after earlier surgery is mainly explained by reduced morbidity of these patients [41, 42].

Other treatment aspects of proximal femoral fractures also affect mortality. Knobe et al. [24] showed that the introduction of a special geriatric treatment pathway alone can improve patient outcome for geriatric patients. The best anesthesia technique for proximal femoral fractures is discussed in recent literature and varies extremely between different countries [43]. Yet, there seems to be no clear evidence favoring general or regional anesthesia according to a recent Cochrane review with regard to mortality [44]. Early mobilization and full weight bearing should be performed after surgery for proximal femoral fracture as it increases functional recovery and reduces complication and mortality rates [45–48]. Latest studies show that liberal transfusion regimens are associated with higher cardiovascular complications in patients with proximal femoral fracture [49, 50]. In our hospital we performed already a restrictive patient blood management as guidelines recommend it. In this study, only the age determined whether a patient was treated interdisciplinary or not. Consequently, patients were included without need for geriatric co-management. Using a test like the ISAR screening tool [51] would enable hospitals to concentrate on patients with need for geriatric co-management. With a more focused patient selection and implementation of geriatric treatment protocols other studies might be able to show improved mortality or complication rates.

Moreover, staff education is another element of orthogeriatric programs [52]. The staff of our orthopedic unit might have improved their knowledge about the diagnosis, prevention and therapy for geriatric syndromes. This gain in knowledge could have improved the therapy for geriatric patients even on days when no geriatrician was involved.

We showed that patients were significantly more satisfied with co-management care. The reasons may be a reduced waiting period for surgery and the more comprehensive care. Boylan et al. showed that higher patient satisfaction is associated with reduced time to surgery and mortality of geriatric femoral neck fracture patients [53]. If we could show increased satisfaction with a less comprehensive model, then satisfaction might also be increased with models that include more frequent geriatric involvement.

### Study limitations and strengths

This study had several limitations. Because the study data were partly extracted from the electronic medical system, our findings and conclusion were limited by the amount of correct and complete documentation. However, we were able to reduce missing values considerable with our follow-up questionnaire.

Furthermore, the follow-up time was sometimes longer than 3 months. Therefore, it might have been difficult for these patients to recall whether they had experienced complications within 3 months after surgery or whether they had been satisfied with the care.

Finally, not all patients could be followed-up at 3 months, due to death or a change in the phone number. This is a known problem in geriatric research.

The study also had some important strengths. To the best of our knowledge, this study was the first to describe the effects of a very simple co-management model with these two additions to the usual care. Our model included only one weekly multidisciplinary round and one reservation in the operation room list for proximal femoral fractures per regular working day. This type of model could be readily implemented in most hospitals in Germany with little effort. Furthermore, this study was the first to report subjective satisfaction rates after proximal femoral fracture treatment in an orthogeriatric setting. We showed that the co-management model significantly improved patient satisfaction.

Future work should focus on the question of which care model would be best for reducing mortality, in addition to improving the functional outcome.

## Conclusion

Our co-management model reduced the LOS and waiting time for surgery. Additionally, it increased patient satisfaction with the care received. Nevertheless, it could not reduce mortality or complication rates during the hospital stay or at a follow-up of at least 3 months. To achieve the latter effects, a more comprehensive model may be needed.

## Data Availability

The datasets used and analyzed during the current study are available from the corresponding author on reasonable request.

## Abbreviations

ADL: Activities of daily living
AHFS: Almelo Hip Fracture Score
ASA score: American Society of Anesthesiologists risk classification score
ICU: Intensive care unit
LOS: Length of hospital stay
TTS: Time to surgery
